# Combined alkylation and histone deacetylase inhibition with EDO-S101 has significant therapeutic activity against brain tumors in preclinical models

**DOI:** 10.18632/oncotarget.25588

**Published:** 2018-06-15

**Authors:** Yushi Qiu, Zhimin Li, John A. Copland, Thomas Mehrling, Han W. Tun

**Affiliations:** ^1^ Department of Cancer Biology, Mayo Clinic, Jacksonville, FL 32224, USA; ^2^ Division of Hematology and Medical Oncology, Mayo Clinic, Jacksonville, FL 32224, USA; ^3^ Mundipharma-EDO GmbH, 4052 Basel, Switzerland

**Keywords:** EDO-S101, brain tumors, CNS lymphoma, glioblastoma multiforme, metastatic breast cancer of the brain

## Abstract

There is a clear unmet need for novel therapeutic agents for management of primary and secondary brain tumors. Novel therapeutic agents with excellent central nervous system (CNS) penetration and therapeutic activity are urgently needed. EDO-S101 is a novel alkylating and histone deacetylase inhibiting agent created by covalent fusion of bendamustine and vorinostat.

We used murine models to perform CNS pharmacokinetic analysis and preclinical therapeutic evaluation of EDO-S101 for CNS lymphoma, metastatic triple-negative breast cancer of the brain, and glioblastoma multiforme. EDO-S101 has excellent CNS penetration of 13.8% and 16.5% by intravenous infusion and bolus administration respectively. It shows promising therapeutic activity against CNS lymphoma, metastatic triple-negative breast cancer of the brain, and glioblastoma multiforme with significant prolongation of survival compared to no-treatment controls. Therapeutic activity was higher with IV infusion compared to IV bolus. It should be evaluated further for therapeutic use in brain tumors.

## INTRODUCTION

Management of primary and secondary brain tumors remains a challenge due to lack of efficacious therapeutic agents with adequate central nervous system (CNS) penetration. Most brain tumors remain incurable with grim prognosis. There is an unmet need for novel therapeutic agents for CNS lymphoma (CNSL), metastatic breast cancer of the brain (MBCB), and glioblastoma multiforme (GBM).

Primary CNSL (PCNSL) is an aggressive diffuse large B-cell lymphoma (DLBCL) mostly of activated B-cell phenotype, which is confined to the CNS [1–4] with an incidence rate of 0.47 per 100,000 person-years [5]. It is an aggressive brain tumor with an average survival time of 1.5 to 3.3 months in untreated patients [6, 7]. The standard current treatment of PCNSL includes induction with high-dose methotrexate-based chemoimmunotherapy followed by consolidation with whole brain radiation, intensive chemotherapy, or high-dose chemotherapy followed by autologous stem cell transplantation [2–4]. These treatments are rather toxic and are not well tolerated, especially by elderly patients in whom the incidence of PCNSL has been rising [2–4]. Once it has relapsed, the prognosis is usually very poor due to limited options for efficacious treatments. Although the survival has improved from a median survival of 12 months 50 years ago to 40% long-term survival at present, it is projected to plateau soon with currently available therapeutic agents [8]. As such, PCNSL remains a devastating brain tumor for which novel therapies are critically needed.

MBCB is a very serious event in the natural history of breast cancer and is associated with very poor prognosis [9, 10]. The incidence of clinically evident brain metastases among women with stage IV breast cancer is 10% to 16% [10]. The true incidence is likely higher as brain metastases are found in 30% of patients at autopsy [10]. Therapy for systemic breast cancer has advanced with development of novel targeted therapeutic agents, resulting in improvement in survival of breast cancer patients. However, the same cannot be said for MBCB. Moreover, there has been an increase in the incidence of MBCB as breast cancer patients are surviving longer [10]. Triple-negative breast cancer is the most aggressive subtype of breast cancer with a high risk for brain metastases [9]. Currently, there is no effective systemic therapy for MBCB [10–12]. Additionally, therapeutic options after failure of radiation therapy are extremely limited [10]. Therapeutic agents with adequate CNS penetration and activity against breast cancer are needed.

The most common and aggressive primary brain tumor is GBM (World Health Organization grade IV astrocytoma) with an incidence rate of 3.19 per 100,000 person-years [13, 14]. In spite of intensive research over the last few decades, impactful progress with improvement in survival has been elusive with the most recent data indicating median survival of 14.6 months [13, 14]. The current therapeutic approach consists of maximal surgical total resection followed by postoperative radiation therapy with concurrent and adjuvant alkylating chemotherapy using temozolomide [14–17]. Response to temozolomide is strongly predicted by methylation status of the promoter of O [6]-methylguanine-DNA methyltransferase (MGMT), a DNA repair enzyme for alkylator-induced DNA damage [14, 18]. Temozolomide does not work well in patients whose tumors are characterized by a lack of MGMT promoter methylation [14, 18]. Bevacizumab, an anti-vascular endothelial growth factor therapy, has not shown great benefit for newly-diagnosed [19, 20] or recurrent [21, 22] GBM.

**Figure 1:**
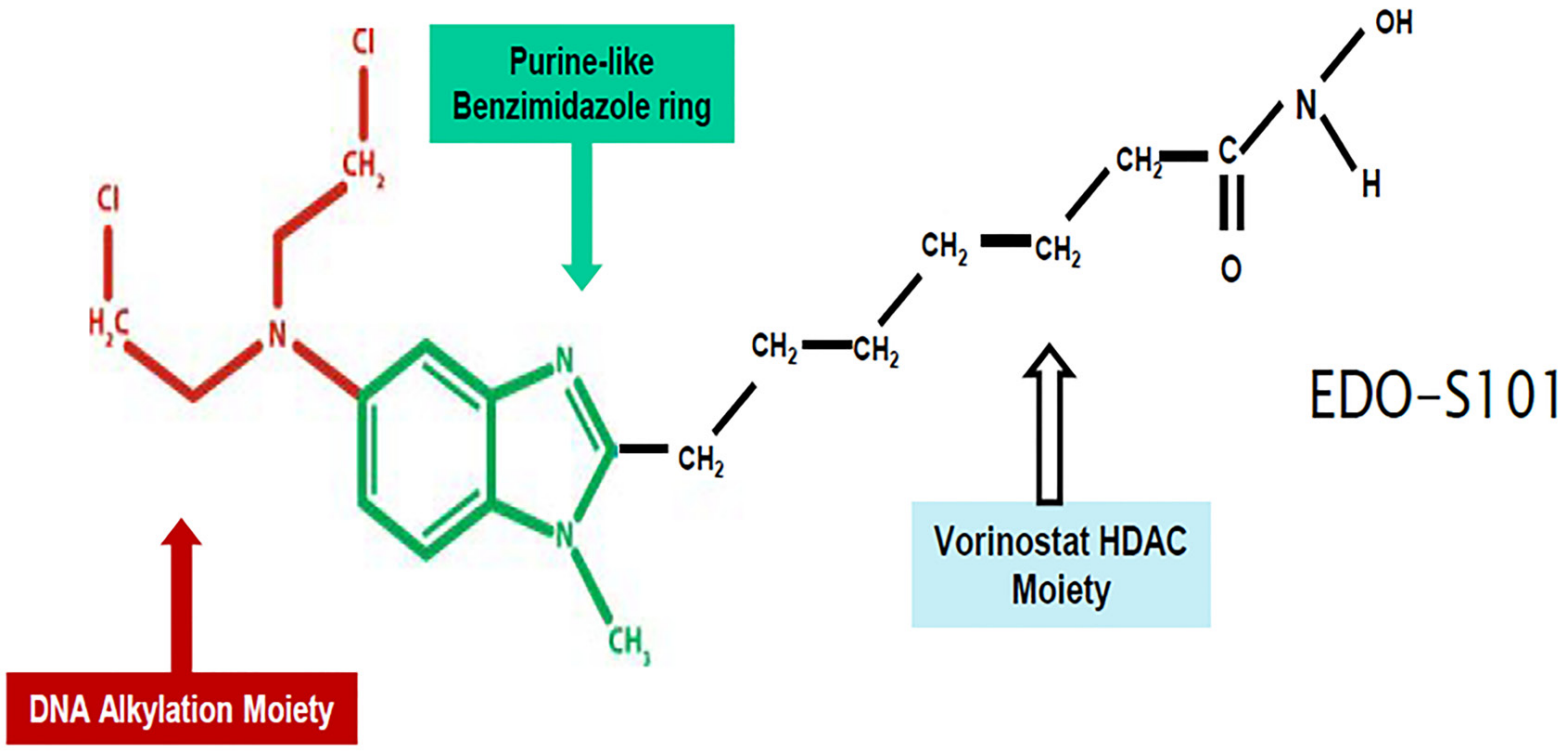
EDO-S101 structure

EDO-S101 is a novel first-in-class fusion molecule of an alkylator, bendamustine, and a histone-deacetylase (HDAC) inhibitor, vorinostat, also known as SAHA [23, 24] (Figure 1). It embodies a novel concept of synergism between alkylation and HDAC inhibition and introduces the alkylating-HDAC inhibition fusion principle [23–28]. Mechanistically, it is a fully bifunctional molecule with retention of alkylating and pan-HDAC inhibitory activities [23, 24]. It induces DNA crosslinking by alkylation, which is 5 times more potent than melphalan and 10 times more potent than bendamustine [23, 24]. Its pan-HDAC inhibitory activity is as strong as vorinostat and is seen at nanomolar concentrations [23, 24]. HDAC inhibitory activity promotes open chromatin conformation via increased acetylation of histones allowing increased accessibility for DNA double strand-breaking by alkylating activity with resultant apoptosis of cancer cells [23, 25–28]. It has been shown to induce apoptosis via intrinsic pathway and cell cycle arrest at G2M phase [23]. It is delivered as a single intact molecule to the cancer cells ensuring generation of alkylation and HDAC inhibition simultaneously in the same location and creating therapeutic synergistic effect [23]. Concomitant exposure to both agents has been shown to be important to achieve maximal therapeutic effect [23, 29]. Pharmacodynamically, concomitant exposure to alkylation and HDAC inhibition cannot be expected to take place simultaneously in a reliable manner if the two agents are administered separately.

The unique mechanistic attributes of EDO-S101 make it a very potent antineoplastic agent, as evidenced by broad-spectrum cytotoxicity against multiple cancer types when tested against the NCI-60 panel of cancer cell lines [23]. EDO-S101 is about 33 times more potent than bendamustine against NCI-60 cell lines with a median IC_50_ of 2.2 uM versus 72.0 uM for bendamustine [23]. Among the cell lines on NCI-60 panels with excellent response to EDO-S101 are GBM and breast cancer cell lines [23]. EDO-S101 has also been tested against hematologic cancer cell lines including lymphoma and leukemia, showing potent cytotoxicity with IC_50_ ranging from 0.4 uM to 5.0 uM [23]. It is currently undergoing a phase 1 clinical trial for relapsed and refractory hematologic malignancies (ClinicalTrials.gov identifier- NCT02576496).

## RESULTS

### CNS pharmacokinetics of EDO-S101

**Figure 2:**
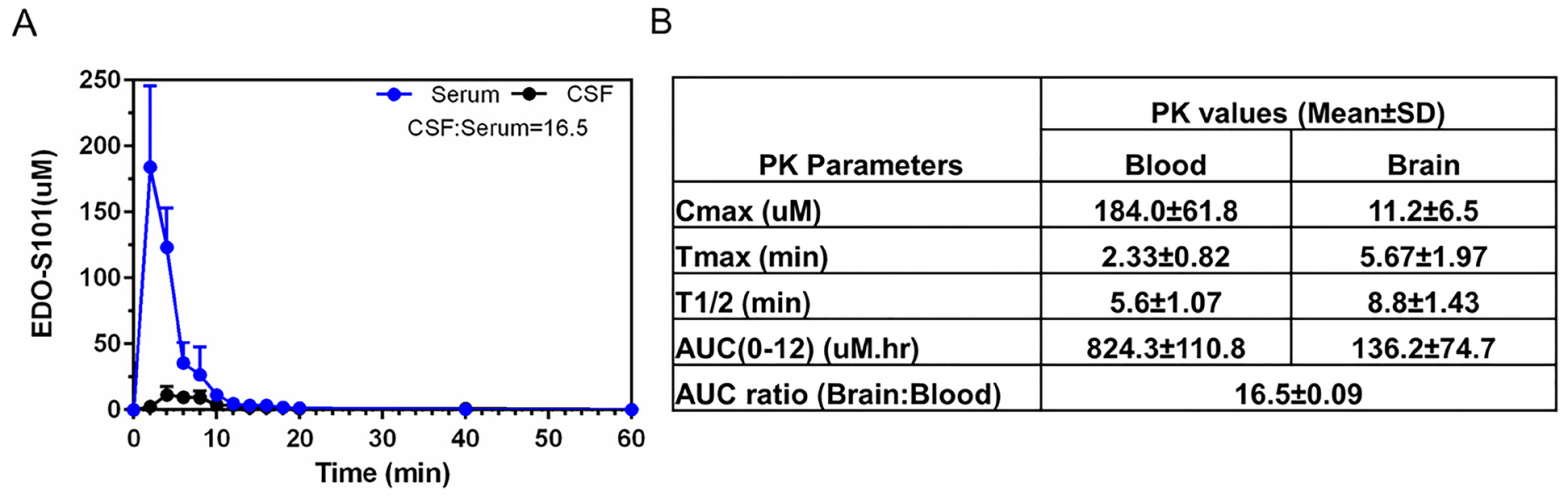
CNS pharmacokinetic analysis of EDO-S101 administered by IV bolus in SD rats (N=6) EDO-S101 40 mg/kg was given IV bolus followed by collection of microdialysates at regular time intervals over 60 minutes from the blood and the brain via microdialysis catheters placed in a carotid artery and a cerebral ventricle. EDO-S101 levels were determined by capillary electrophoresis. **(A)** Time concentration curves of EDO-S101 in the blood and the brain are shown. **(B)** Pharmacokinetic parameters of EDO-S101 in the blood and the brain are shown. CNS penetration of EDO-S101 is ~16.5 as calculated by the AUC ratio of brain and blood.

**Figure 3:**
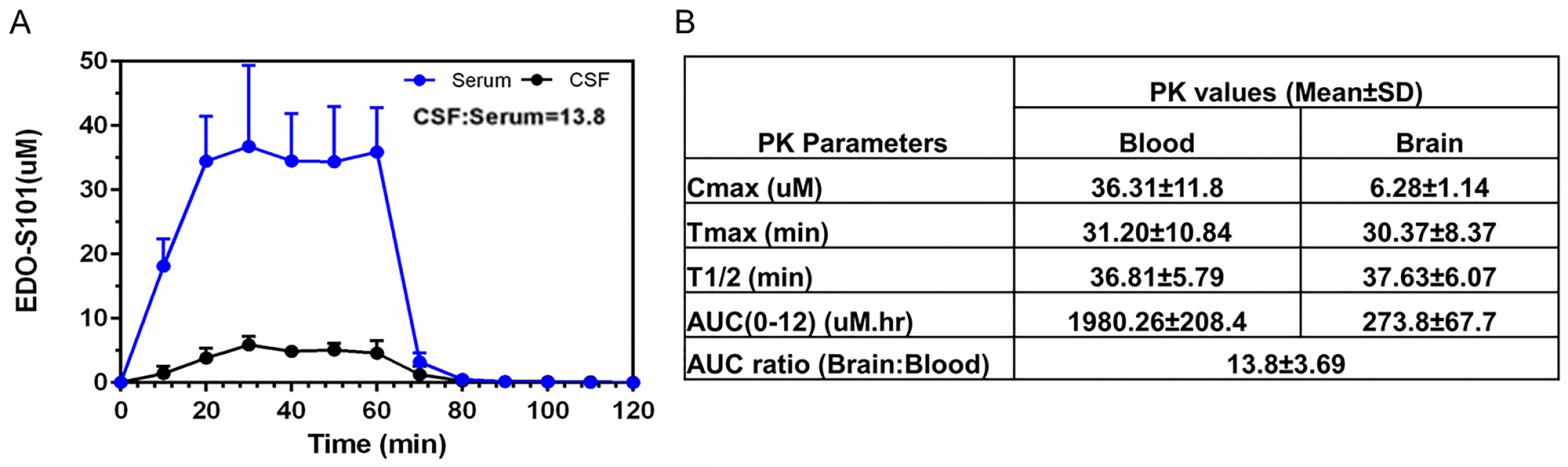
CNS pharmacokinetic analysis of EDO-S101 administered by one hour IV infusion in C57BL/6J (N=6) EDO-S101 30 mg/kg was given by IV infusion over one hour followed by collection of microdialysates at regular time intervals over 120 minutes from the blood and the brain via microdialysis catheters placed in a carotid artery and a cerebral ventricle. EDO-S101 levels were determined by capillary electrophoresis. **(A)** Time concentration curves of EDO-S101 in the blood and the brain are shown. **(B)** Pharmacokinetic parameters of EDO-S101 in the blood and the brain are shown. CNS penetration of EDO-S101 is ~13.8 as calculated by the AUC ratio of brain and blood.

Excellent CNS penetration with adequate therapeutic CNS concentration is an essential prerequisite of antineoplastic agents for the treatment of brain tumors. We performed a CNS pharmacokinetic analysis on EDO-S101 administered by 2 schedules, IV bolus 40 mg/kg in SD rats and CIVI 30 mg/kg over one hour in C57BL/6J mice. Pharmacokinetic findings are presented in Figure 2 and Figure 3. EDO-S101 was cleared quickly from both the blood and the brain with a short half-life. It can cross the blood-brain barrier with CNS penetration of 16.5% and 13.8% for IV bolus and CIVI administrations, respectively. The maximum concentration in the brain achieved by both administration methods was higher than the median IC_50_ value of 2.2 uM for NCI-60 cancer cell lines and IC_50_ values of 0.4 uM to 5.0 uM for hematologic malignancy cancer cell lines [23], indicating that EDO-S101 achieves adequate therapeutic concentration in the brain. The CNS pharmacokinetic findings indicate that EDO-S101 is a good candidate for assessment of therapeutic activity against brain tumors.

### Therapeutic activity of EDO-S101 against CNSL in OCI-LY10 murine model

PCNSL is a DLBCL confined to the CNS [2, 4]. Most of the patients with CNSL cannot be cured with the current treatments, which are rather toxic and not well tolerated [2, 4]. As such, there is a need for novel therapeutic agents. Bendamustine has shown antilymphoma activity and is currently used as a chemotherapeutic agent for various types of lymphoma including indolent non-Hodgkin lymphoma, mantle cell lymphoma, DLBCL, and Hodgkin lymphoma [30]. Vorinostat has also shown antilymphoma activity, especially against cutaneous T cell lymphoma [31–33]. EDO-S101 has shown potent cytotoxic activity against DLBCL [23]. In a study on diffuse large B cell lymphoma cell lines, the concomitant treatment with bendamustine and vorinostat showed enhanced histone acetylation and double strand DNA breaks resulting in an additive to synergistic cytotoxic effect in both ABC- and GCB-type DLBCL cells independent of p53 mutation status [29]. However, improved cytotoxicity is not seen when lymphoma cells are treated sequentially [29].

Therapeutic activity of EDO-S101 against CNSL was tested in a murine model created by intracerebral implantation in athymic mice of OCI-LY10 DLBCL cells, which are the same subtype of DLBCL as PCNSL (activated B-cell subtype). EDO-S101 was administered by repeat IV bolus and repeat CIVI schedules.

**Figure 4:**
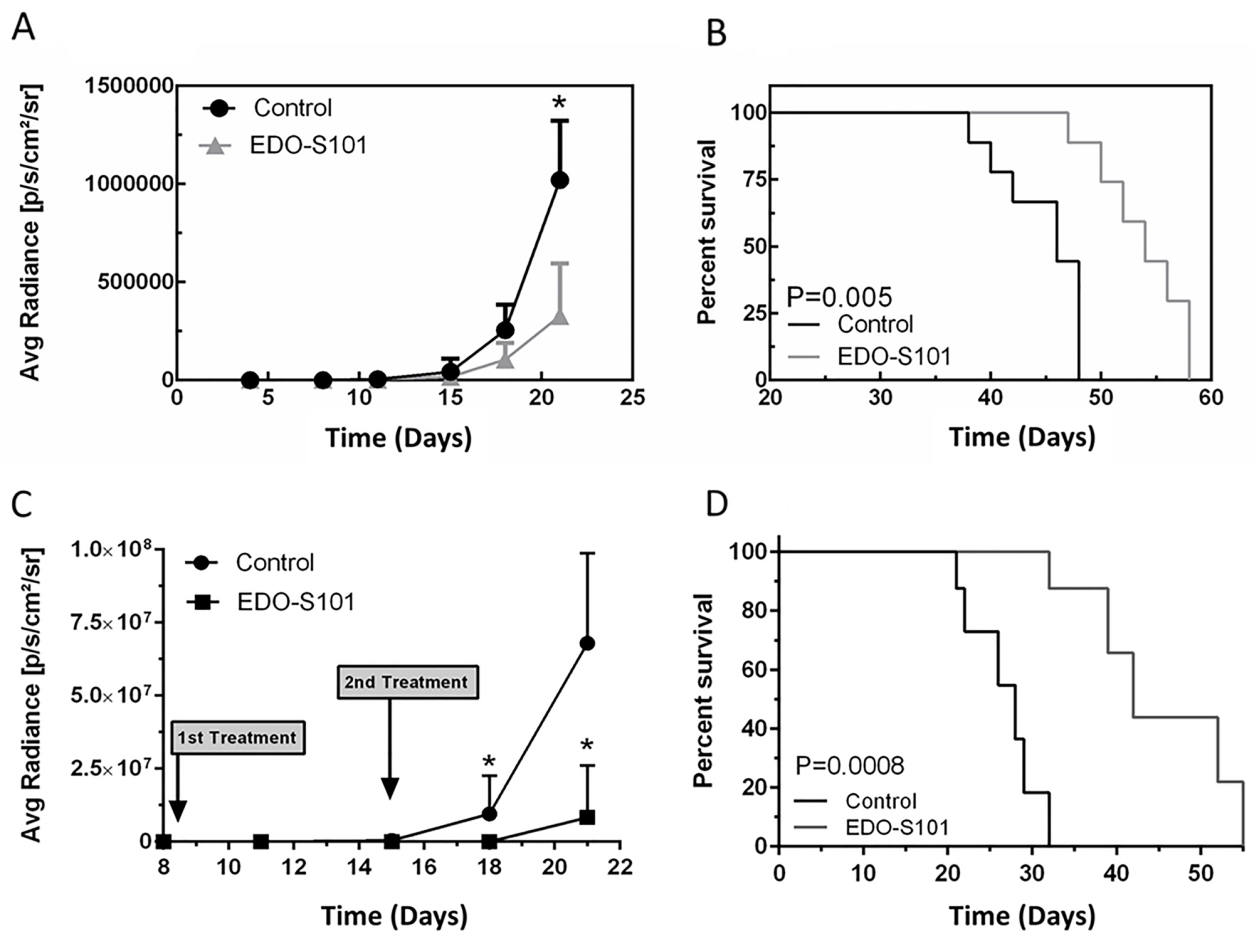
EDO-S101 has significant therapeutic activity against CNS lymphoma Therapeutic activity of EDO-S101 against CNS lymphoma was tested in OCI-LY10 murine model. 5 × 10^5^ luciferase-transfected OCI-LY10 lymphoma cells were intracerebrally injected in L periventricular area in athymic mice. Therapeutic activity was assessed by impact on tumor growth as reflected by bioluminescence activity (A & C) and survival analysis (B & D). Mice in control group received DMSO on the similar treatment schedule as EDO-S101 treatment group. N= 10 in treatment and control groups. **(A & B)** Repeat IV bolus administration of EDO-S101. The treatments were given on days 4, 11, and 18 post tumor implantation. **(C & D)** Repeat continuous IV infusion of EDO-S101. The treatments were given on days 8 and 15 post tumor implantation by IV infusion over one hour via an infusion pump. EDO-S101 treatment resulted in suppression of tumor growth which translated into significant prolongation of survival (P<0.05).

Both treatment schedules showed significant therapeutic activity with suppression of tumor growth and prolongation of survival compared to the dimethyl sulfoxide (DMSO) control group (Figure 4). Median survival was 54 days in the EDO-S101 by repeat IV bolus treatment group vs 46 days in the DMSO control group, whereas it was 60 days in the repeat CIVI treatment group vs 49 days in the control group. EDO-S101 treatment appeared to be better tolerated with the CIVI schedule compared to the IV bolus schedule based on observation of clinical toxicities including changes in weight, mobility, and feeding.

### Therapeutic activity of EDO-S101 against triple-negative MBCB in MB-468 murine model

MBCB carries a very poor prognosis as no efficacious treatments are currently available [10]. Novel therapeutic agents that produced substantial improvement in non-CNS disease unfortunately do not cross the blood-brain barrier well and have not had any major impact on prognosis of CNS disease [10]. EDO-S101 showed significant cytotoxicity against breast cancer cell lines in the NCI-60 panel (MCF7, BT-549, MDA-MB 231, T47D, MDA-MB-468, and HS578T) [23]. BT-549, MDA-MB 231, MDA-MB-468, and HS578T are triple negative breast cancer cell lines on the panel and showed significant sensitivity to EDO-S101 [23]. Among subtypes of breast cancer, triple-negative breast cancer is the most aggressive and has predilection for metastasizing to the brain [9].

**Figure 5:**
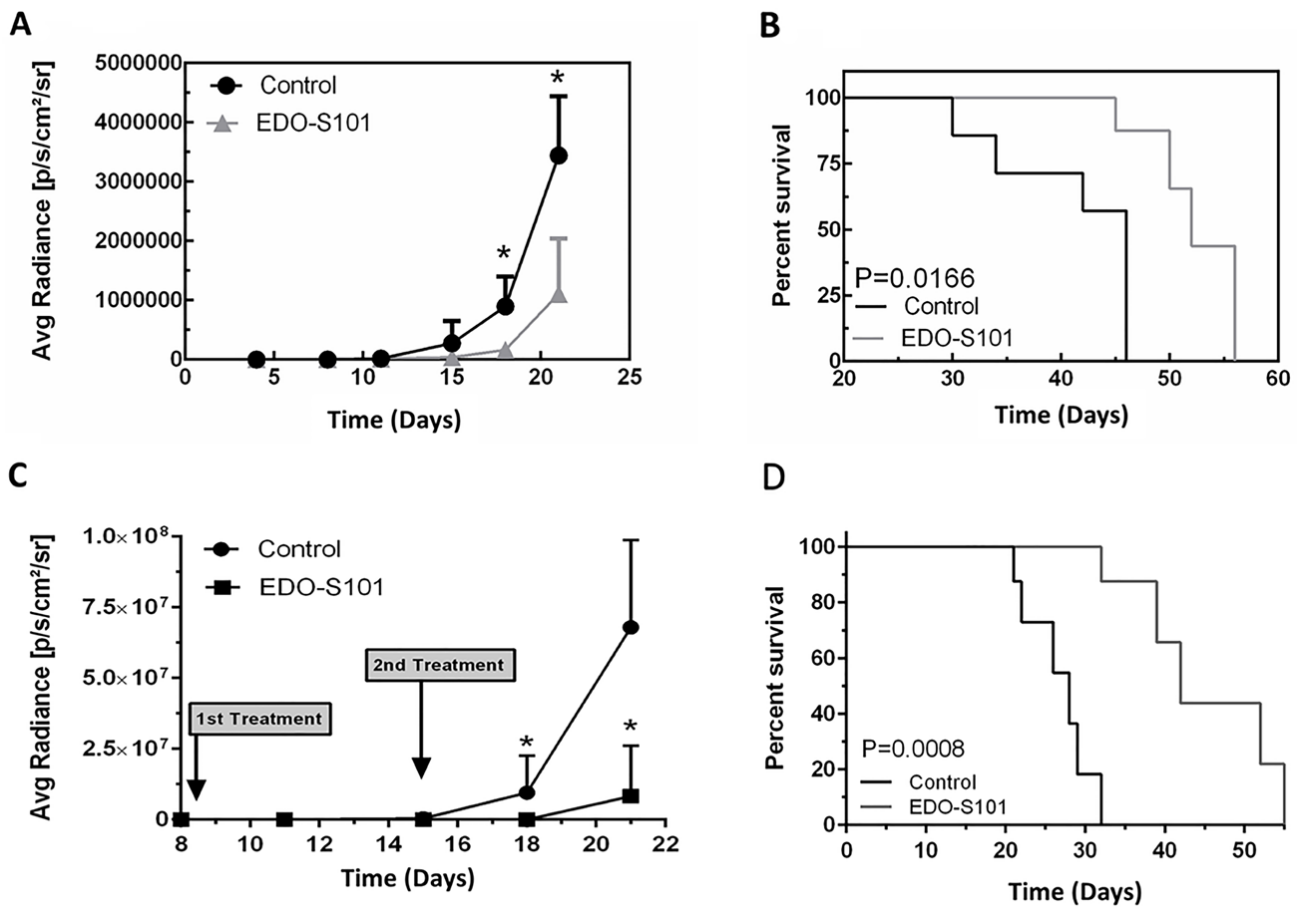
EDO-S101 has significant therapeutic activity against metastatic breast cancer of the brain Therapeutic activity of EDO-S101 against metastatic breast cancer of the brain was tested in MB-468 murine model. 5 × 10^5^ luciferase-transfected MB-468 triple negative breast cancer cells were intracerebrally injected in L periventricular area in athymic mice. Therapeutic activity was assessed by impact on tumor growth as reflected by bioluminescence activity (A & C) and survival analysis (B & D). Mice in control group received DMSO on the similar treatment schedule as EDO-S101 treatment group. N= 10 in treatment and control groups. **(A & B)** Repeat IV bolus administration of EDO-S101. The treatments were given on days 4, 11, and 18 post tumor implantation. **(C & D)** Repeat continuous IV infusion of EDO-S101. The treatments were given on days 8 and 15 post tumor implantation by IV infusion over one hour via an infusion pump. EDO-S101 treatment resulted in suppression of tumor growth which translated into significant prolongation of survival (P<0.05).

Therapeutic activity of EDO-S101 against triple-negative MBCB was tested in a murine model created by intracerebral implantation of triple-negative MDA-MB-468 breast cancer cells in athymic mice. Both repeat IV bolus and repeat CIVI treatment schedules showed significant therapeutic activity with suppression of tumor growth and prolongation of survival compared to DMSO control group (Figure 5). Median survival was 52 days in the EDO-S101 by repeat IV bolus treatment group vs 46 days in the DMSO control group, whereas it was 42 days in the repeat CIVI treatment group vs 27 days in the control group. EDO-S101 treatment appeared to be better tolerated with the CIVI schedule compared to the IV bolus schedule based on clinical observation.

### Therapeutic activity of EDO-S101 against GBM in a patient-derived xenograft model

GBM is the most common and aggressive primary brain tumor [14]. In spite of intensive research efforts, GBM remains incurable [13, 14]. Novel efficacious therapeutic agents are needed to fill this unmet therapeutic void. The standard chemotherapeutic agent for GBM is an alkylating agent, temozolomide [34, 35]. HDAC inhibition with vorinostat has shown therapeutic activity against GBM in a clinical trial [36]. As such, EDO-S101 with bifunctional activity is a promising therapeutic agent for GBM from a mechanistic standpoint. EDO-S101 showed notable *in vitro* cytotoxicity against GBM cell lines on NCI-60 panel (SF-268, SF-295, SF-539, SNB-19, SNB-75, and U-251), showing activity against GBM cell lines with both methylated and unmethylated MGMT promotor [23].

**Figure 6:**
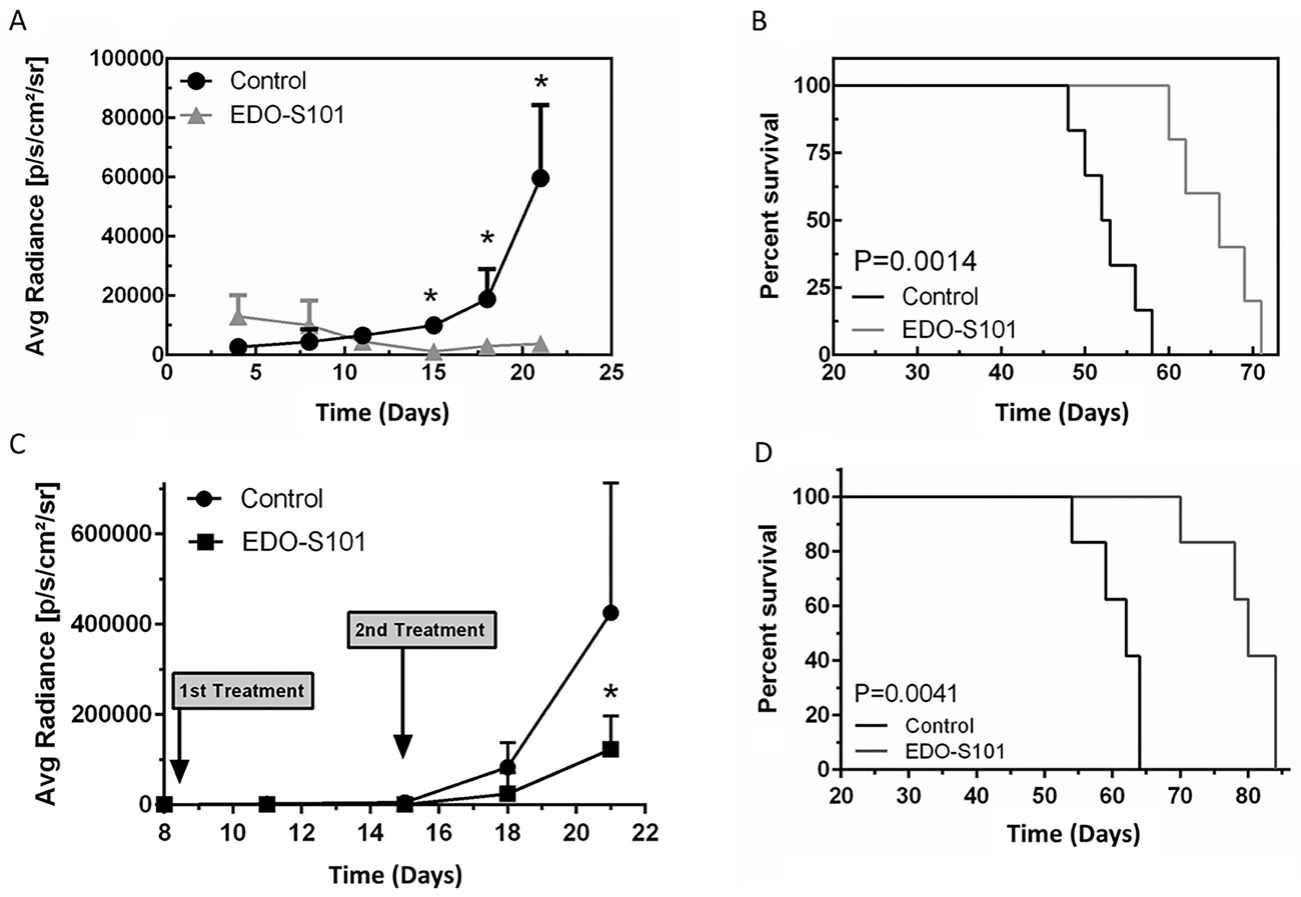
EDO-S101 has significant therapeutic activity against glioblastoma multiforme Therapeutic activity of EDO-S101 against glioblastoma multiforme was tested in GBM12 patient derived xenograft model. 5 × 10^5^ luciferase-transfected GBM12 cells were intracerebrally injected in L periventricular area in athymic mice. Therapeutic activity was assessed by impact on tumor growth as reflected by bioluminescence activity (A & C) and survival analysis (B & D). Mice in control group received DMSO on the similar treatment schedule as EDO-S101 treatment group. N= 10 in treatment and control groups. **(A & B)** Repeat IV bolus administration of EDO-S101. The treatments were given on days 4, 11, and 18 post tumor implantation. **(C & D)** Repeat continuous IV infusion of EDO-S101. The treatments were given on days 8 and 15 post tumor implantation by IV infusion over one hour via an infusion pump. EDO-S101 treatment resulted in suppression of tumor growth which translated into significant prolongation of survival (P<0.05).

Therapeutic activity of EDO-S101 against GBM was tested in a GBM12 patient-derived xenograft model. Biologically, GBM12 cells show methylation of the promoter of MGMT and sensitivity to temozolomide [37]. Both repeat IV bolus and repeat CIVI treatment schedules showed significant therapeutic activity with suppression of tumor growth and prolongation of survival compared to the DMSO control group (Figure 6). Median survival was 62 days in the EDO-S101 by repeat IV bolus treatment group vs 52 days in the DMSO control group, whereas it was 80 days in the repeat CIVI treatment group vs 63 days in the control group. Similar to CNSL and MBCB experiments, EDO-S101 treatment appeared to be better tolerated with the CIVI schedule compared to IV bolus schedule based on observation of clinical toxicities.

## DISCUSSION

Our results indicate that EDO-S101 is a novel CNS-penetrating antineoplastic agent with impressive preclinical therapeutic activity against 3 aggressive brain tumors (CNSL, MBCB, and GBM). Our CNS pharmacokinetic analysis showed excellent CNS penetration, which enables it to achieve adequate therapeutic CNS concentration. Its maximum concentration in the blood and brain are substantially higher than its median IC_50_ value for NCI-60 cell lines and IC_50_ values for hematologic cancer cell lines. As such, it likely has broad-spectrum therapeutic activity against primary and secondary brain tumors. Bifunctional mechanistic activity of EDO-S101 with synergism between simultaneous alkylation and HDAC inhibition taking place in the cancer cells may explain its unique therapeutic activity.

Currently, there is an unmet therapeutic need for the 3 brain tumors for which we tested EDO-S101 in preclinical models. Primary and secondary CNSLs remain mostly incurable with currently available treatments, which are rather toxic and not well tolerated [2–4]. In a small retrospective study in patients with recurrent PCNSL refractory to high-dose methotrexate, bendamustine was shown to have modest single-agent activity with manageable toxicity [38]. EDO-S101 should be evaluated further in CNSL.

Although great strides have been made over the last few decades leading to improvement in the prognosis of systemic breast cancer, very little improvement has been seen for MBCB. This grim situation exists mainly because the novel therapeutic agents developed over the last few decades do not adequately penetrate the brain. Excellent CNS penetration of EDO-S101 combined with our *in vivo* results and excellent cytotoxicity against NCI-60 breast cancer cell lines are encouraging.

The preclinical therapeutic findings of EDO-S101 against GBM are quite exciting. Alkylation (temozolomide) [34, 35] and HDAC inhibition (vorinostat) [36] have been associated with therapeutic activity in GBM. However, a recent phase I/II trial of vorinostat combined with temozolomide and radiation therapy for newly diagnosed glioblastoma did not demonstrate significant activity [39]. We have to note that vorinostat and temozolomide administered separately cannot be considered as equivalent to EDO-S101, which is a fusion drug consisting of bendamustine and vorinostat. Moreover, EDO-S101 has been shown to have activity against temozolomide-sensitive as well as resistant GBM cell lines [23]. As such, the clinical trial findings cannot be used to predict therapeutic efficacy of EDO-S101. As EDO-S101 has a unique synergistic bifunctionality of alkylation and HDAC inhibition, it represents a new therapeutic approach for GBM and should be explored further in other preclinical GBM models representing various genetic and molecular subsets with the ultimate aim of translating into a phase 1 clinical trial.

In this study, we tested 2 treatment schedules, repeat IV bolus and repeat 1-hour CIVI. Both administration schedules were associated with therapeutic activity. The total dose of EDO-S101 administered was 60 mg/kg and 180 mg/kg for CIVI and IV bolus schedules, respectively. In spite of this degree of difference in dose intensity, considerable therapeutic activity was still observed with the CIVI schedule. The CIVI schedule appears to be better tolerated based on our observation of clinical toxicity in animals, indicating that there is still a lot of room for dose intensification for this treatment schedule. As such, the CIVI schedule appears to be more suitable for further evaluation.

In conclusion, EDO-S101 holds promise for the treatment of primary and secondary brain tumors and should be further evaluated in clinical trials.

## MATERIALS AND METHODS

### CNS pharmacokinetic analysis

CNS pharmacokinetic analysis was performed in murine models to determine the CNS penetration of EDO-S101. EDO-S101 was administered by single-dose intravenous (IV) bolus (40 mg/kg) in SD rats or continuous IV infusion (CIVI; 30 mg/kg) for 1 hour via an infusion pump in C57BL/6J mice. Microdialysates were collected at regular time intervals from the brain or blood via microdialysis catheters in a ventricle of the brain and a carotid artery. Concentration of the drug in the microdialysate samples is determined by capillary electrophoresis with ultraviolet detection at 280 nM. Details of microdialysis and capillary electrophoresis were previously published [40]. Time-concentration graphs were constructed and CNS pharmacokinetic parameters were calculated. CNS penetration of the drug is calculated as the percent area under the curve ratio of brain and blood.

### Preclinical brain tumor models

Details of intracerebral implantation of tumor cells were previously published [41]. The CNSL model was created by intracerebral injection of luciferase-transfected OCI-LY10 DLBCL cells into the left periventricular area of the brain in athymic mice. The cells were provided by Arthur L. Shaffer, III, National Cancer Institute of Health, Bethesda, Maryland. The glioblastoma patient-derived xenograft model was created by intracerebral injection of luciferase-transfected primary GBM cells (GBM12) into left periventricular area of the brain in athymic mice. The cells were provided by Dr. Jan Sakaria, Mayo Clinic, Rochester, Minnesota [37]. The MBCB model was created by intracerebral injection of luciferase-transfected MB-468 triple-negative breast cancer cells into left periventricular area of the brain in athymic mice. The cells were purchased from ATCC (Manassas, Virginia).

### Treatment with EDO-S101

In the IV bolus group, 60 mg/kg of EDO-S101 was administered by repeat IV bolus via tail vein injection on days 4, 11, and 18 after tumor implantation. In the CIVI group, 30 mg/kg of EDO-S101 was administered by repeat CIVI via an infusion port over 1 hour on days 8 and 15 after tumor implantation.

### Bioluminescence imaging

Bioluminescence imaging was used to study the impact of treatment on intracerebral tumor growth in real time. The detailed methodology has been previously published [41].

### Statistics

Analysis of variance was used to determine statistical significance of the differences between experimental groups. Survival analysis was performed by Kaplan-Meier method using limb paralysis as the end point. Kaplan-Meier survival curves were generated using Prism4 software (GraphPad Software, LaJolla, CA), and the statistical difference between curves was derived with a log-rank test. *P*<.05 was considered significant.

## Abbreviations

CNS: Central nervous system
MBCB: Metastatic breast cancer of the brain
GBM: Glioblastoma multiforme
DLBCL: Diffuse large B-cell lymphoma
MGMT: O [6]-methylguanine-DNA methyltransferase
HDAC: Histone-deacetylase
CIVI: Continuous IV infusion
DMSO: Dimethyl sulfoxide
